# Supplementary material for: Quantitative use-wear analysis of stone tools: Measuring how the intensity of use affects the identification of the worked material
Source: PLoS One. 2021 Sep 20;16(9):e0257266. doi: 10.1371/journal.pone.0257266 (PMC8452086; doi:10.1371/journal.pone.0257266)
Supplement: Attachment — Submitted filename: POINT BY POINT ANSWER.docx [file pone.0257266.s003.docx]

POINT BY POINT ANSWER

This paper is a highly specialised methodological study, whose title and abstract are only comprehensible to a small group of specialists. If you wish it to be considered for publication in PLOS ONE you will need to revise the title, to make is more broadly explanatory. You will also need to provide a broader contextualisation and include features of this is your abstract; this needs to be comprehensible to a wide scientific audience. Currently, even as someone who does understand the context, I am still unaware after reading the Abstract and looking at the title, whether you are referring to stone tools, or archaeological tools in general, for example. You mention ‘lithics’ somewhere in the middle of your Introduction, so when I reached this point, I was finally able to understand that you are referring specifically to ‘stone tools’ or lithics. You also provide no explanation as to why it is important to understand worked materials, nor do you explain what these are, in other words, what is the value of microwear analysis? Currently, the Supplementary Data files are incomprehensible; they need to be accompanied by full explanations.

We have changed the title of the paper and the abstract in order to make it more broadly explanatory. We have added detailed explanations for the Supplementary Data files.

I also wonder what ‘Nouse’ is? (Tables 3, 4). Going further down your text I wonder if you mean ‘no use’ I suggest you replace this with ‘unused’.

We have changed Nouse by Unused

My comments in no way reflect any issues with the scientific content of your manuscript, rather I am acting as a Devil’s Advocate, to give you the opportunity to revise and re-submit, in a way that would make your clearly important study, more attractive for publication in PLOS ONE, which is a broad scientific journal. In order for it to be suitable for consideration, you need to redesign the way you present it, so that the title and abstract are more explanatory to a wider audience and you need to provide a broader contextualisation in your Introduction to explain why it is important to study ‘worked materials’ and indeed, what these are. I suggest you change the title, revise your abstract and provide a broader Introduction and explanatory background before resubmitting.
