# Supplementary material for: Quantitative use-wear analysis of stone tools: Measuring how the intensity of use affects the identification of the worked material
Source: PLoS One. 2021 Sep 20;16(9):e0257266. doi: 10.1371/journal.pone.0257266 (PMC8452086; doi:10.1371/journal.pone.0257266)
Supplement: Attachment — Submitted filename: Rensponse to reviewers.docx [file pone.0257266.s005.docx]

We have corrected the references as suggested by reviewer 2
